# Identification of genes specifically required for the anaerobic metabolism of benzene in *Geobacter metallireducens*

**DOI:** 10.3389/fmicb.2014.00245

**Published:** 2014-05-22

**Authors:** Tian Zhang, Pier-Luc Tremblay, Akhilesh K. Chaurasia, Jessica A. Smith, Timothy S. Bain, Derek R. Lovley

**Affiliations:** ^1^Department of Microbiology, University of MassachusettsAmherst, MA, USA; ^2^The Novo Nordisk Foundation Center for Biosustainability, Technical University of DenmarkHørsholm, Denmark

**Keywords:** benzene activation, anaerobic oxidation, phenol, *Geobacter metallireducens*, oxidoreductase

## Abstract

Although the biochemical pathways for the anaerobic degradation of many of the hydrocarbon constituents in petroleum reservoirs have been elucidated, the mechanisms for anaerobic activation of benzene, a very stable molecule, are not known. Previous studies have demonstrated that *Geobacter metallireducens* can anaerobically oxidize benzene to carbon dioxide with Fe(III) as the sole electron acceptor and that phenol is an intermediate in benzene oxidation. In an attempt to identify enzymes that might be involved in the conversion of benzene to phenol, whole-genome gene transcript abundance was compared in cells metabolizing benzene and cells metabolizing phenol. Eleven genes had significantly higher transcript abundance in benzene-metabolizing cells. Five of these genes had annotations suggesting that they did not encode proteins that could be involved in benzene metabolism and were not further studied. Strains were constructed in which one of the remaining six genes was deleted. The strain in which the monocistronic gene Gmet 0232 was deleted metabolized phenol, but not benzene. Transcript abundance of the adjacent monocistronic gene, Gmet 0231, predicted to encode a zinc-containing oxidoreductase, was elevated in cells metabolizing benzene, although not at a statistically significant level. However, deleting Gmet 0231 also yielded a strain that could metabolize phenol, but not benzene. Although homologs of Gmet 0231 and Gmet 0232 are found in microorganisms not known to anaerobically metabolize benzene, the adjacent localization of these genes is unique to *G. metallireducens*. The discovery of genes that are specifically required for the metabolism of benzene, but not phenol in *G. metallireducens* is an important step in potentially identifying the mechanisms for anaerobic benzene activation.

## Introduction

Microbial degradation of the hydrocarbons in petroleum reservoirs is of interest because of its potential impact on the hydrocarbon composition and quality of deposits as well as reservoir souring (Head et al., 2010). Furthermore, anaerobic oxidation of hydrocarbons coupled to the reduction of Fe(III) minerals can have a significant influence on subsurface biogeochemistry, including the production of magnetite which can provide magnetic anomaly signals that can aid in localizing deposits (Lovley, 1991).

The availability of pure cultures capable of anaerobically degrading alkanes and substituted aromatic hydrocarbons has yielded a substantial understanding of the pathways for the degradation of these constituents of crude oil (Widdel and Rabus, 2001; Foght, 2008; Meckenstock and Mouttaki, 2011; Heider and Schühle, 2013). Due to a paucity of model pure cultures, less is known about the anaerobic degradation of benzene, a significant crude oil constituent, not only because of its value as a petrochemical/fuel component, but also because of its human toxicity (Lovley, 2000; Coates et al., 2002; Vogt et al., 2011).

Until recently, the study of anaerobic benzene degradation has been limited to investigations with mixed cultures with a focus on the ability of anaerobes to remove benzene from contaminated groundwater (Widdel and Rabus, 2001; Foght, 2008; Lovley et al., 2011; Meckenstock and Mouttaki, 2011; Heider and Schühle, 2013). Anaerobic benzene degradation has been documented under methanogenic conditions (Grbic-Galic and Vogel, 1987; Weiner and Lovley, 1998; Sakai et al., 2009; Masumoto et al., 2012) as well as with either Fe(III) (Lovley et al., 1994, 1996), sulfate (Lovley et al., 1995; Anderson and Lovley, 2000), nitrate (van der Zaan et al., 2012), or an electrode (Zhang et al., 2010) serving as the electron acceptor. In some of these mixed culture studies there was evidence that the first step in anaerobic benzene activation was conversion to phenol (Vogel and Grbicgalic, 1986; Grbic-Galic and Vogel, 1987; Weiner and Lovley, 1998; Caldwell and Suflita, 2000), whereas in other instances there was evidence that benzene was first metabolized to benzoate (Chaudhuri and Wiesmann, 1995; Caldwell and Suflita, 2000; Kunapuli et al., 2008; Abu Laban et al., 2010) or toluene (Ulrich et al., 2005). However, technical difficulties in working with mixed cultures have prevented definitive studies on the mechanisms for anaerobic benzene activation.

*Dechloromonas aromatica* grew with benzene as the sole electron donor in an anaerobic medium with nitrate as the electron acceptor (Coates et al., 2001; Chakraborty and Coates, 2005; Chakraborty et al., 2005). Phenol was proposed to be the first product of benzene activation. However, the finding that the oxygen in the phenol produced was not derived from water (Chakraborty and Coates, 2005), as well as the presence of genes for oxygen-dependent benzene metabolism coupled with a lack of genes for anaerobic metabolism of phenol or other potential aromatic intermediates (Salinero et al., 2009), suggested that molecular oxygen was involved in benzene metabolism, even though the medium was anaerobic. It has been suggested that *D. aromatica* utilizes molecular oxygen generated intracellularly from nitrate for benzene activation (Salinero et al., 2009; Weelink et al., 2010; Meckenstock and Mouttaki, 2011; Vogt et al., 2011), but this possibility has yet to be experimentally verified.

The hyperthermophilic archeon, *Ferroglobus placidus* was the first organism in pure culture unequivocally found to be capable of anaerobically oxidizing benzene (Holmes et al., 2011). *F. placidus* oxidizes benzene to carbon dioxide with Fe(III) as the sole electron acceptor (Holmes et al., 2011). Benzoate, but not phenol or toluene, transiently accumulated during benzene metabolism and [^14^C]-benzoate was produced from [^14^C]-benzene (Holmes et al., 2011). Genome-scale transcriptional analysis demonstrated that during growth on benzene, there was an increase in transcript abundance for genes specifically involved in the metabolism of benzoate, but not phenol. Transcript abundance for a putative carboxylase gene was higher during growth on benzene vs. benzoate, suggesting a potential candidate enzyme for the carboxylation reaction (Holmes et al., 2011). The lack of a genetic system for *F. placidus*, and the technical difficulties of working with a hyperthermophile, has limited further investigation of the benzene degradation pathway in *F. placidus*.

However, it has subsequently been found that *Geobacter metallireducens*, which can be genetically manipulated (Oberender et al., 2012; Tremblay et al., 2012; Smith et al., 2013), is also capable of anaerobically oxidizing benzene with the reduction of Fe(III) (Zhang et al., 2012, 2013). As previously reviewed (Lovley et al., 2011), *Geobacter* species are thought to be important agents for the removal of benzene and other aromatic hydrocarbons from a diversity of contaminated subsurface environments in which Fe(III) minerals are available. Multiple lines of evidence demonstrated that *G. metallireducens* metabolized benzene via a phenol intermediate rather than benzoate (Zhang et al., 2013). For example, small amounts of phenol were detected during growth on benzene and ^18^O-labeling studies demonstrated that oxygen was derived from water to generate phenol. Transcripts for genes specifically involved in the metabolism of phenol were more abundant during growth on benzene than during growth on alternative aromatic substrates, and the deletion of the genes for subunits of two enzymes involved in phenol degradation prevented the metabolism of benzene whereas deleting genes specific for benzoate or toluene metabolism had no impact on benzene metabolism.

The conversion of benzene to phenol is an exergonic reaction at pH 7 under standard conditions:

$$\text{Benzene}+{\text{H}}_{2}O\to \text{phenol}+2{\text{H}}^{+}\Delta {\text{G}}^{0^{\prime }}=-14.7\text{kJ/mol}$$

          (calculated with data from Thauer et al., 1977)

The exergonic hydroxylation reaction of benzene by *G. metallireducens* is consistent with other characterized biological anaerobic hydroxylation reactions such as the conversion of ethylbenzene to (*S*)-1-phenylethanol and the hydration of acetylene to acetaldehyde, which are exergonic reactions performed by enzymes belonging to oxidoreductase families (Rosner and Schink, 1995; Johnson et al., 2001; Kniemeyer and Heider, 2001).

Here we report on studies designed to identify genes encoding enzymes involved in the initial activation of benzene to phenol. Target genes identified from previously published (Zhang et al., 2013) whole-genome transcriptomic analysis were deleted to ascertain genes required for the metabolism of benzene, but not phenol, that might encode candidate enzymes for benzene activation.

## Materials and methods

### Organisms and culture conditions

The bacterial strains and plasmids used in this study are listed in Table S1. *Geobacter metallireducens* (ATCC 53774 and DSM 7210) (Lovley et al., 1993) was routinely cultured under strict anaerobic conditions at 30°C with acetate (10 mM) as the electron donor and Fe(III) citrate (50 mM) as the electron acceptor, as previously described (Lovley and Phillips, 1988).

Metabolism of labeled compounds was investigated with cell suspensions as previously described (Zhang et al., 2013). Additions for [^14^C]-labeling studies were 2.59 × 10^5^ Bq of ^14^C [39 μM [UL-^14^C]benzene (2.78 × 10^9^ Bq mmol^−1^; Moravek Biochemicals, Brea, CA, USA) or 1.85 × 10^5^ Bq [U-^14^C]phenol (2.96 × 10^9^ Bq mmol^−1^; ARC, St-Louis, MO, USA)].

### Analysis of gene expression

Whole-genome microarray analysis of gene transcript abundance in cells metabolizing different aromatic substrates or acetate have previously been described (Zhang et al., 2013; NCBI GEO under accession number GSE33794). The microarray results were analyzed with Array 4 Star (DNASTAR, Madison, WI, USA).

### Mutant construction

Mutants were constructed as described previously (Zhang et al., 2013). Genomic DNA was extracted with the Epicentre MasterPure DNA Purification Kit (Epicentre Biotechnologies, Madison, WI, USA). Plasmids were extracted with the QIAprep Spin Miniprep Kit (Qiagen, Valencia, CA, USA). PCR amplification was done with the Taq polymerase (Qiagen). DNA gel purification was done with the QIAquick gel extraction kit (Qiagen). Mutant alleles were constructed by replacing the coding sequences with a spectinomycin resistance. Briefly, upstream and downstream flanking regions of the genes to be deleted (ca. 500 bp) were amplified. PCR products were mixed, digested with *AvrII* (NEB, Beverly, MA) and ligated with the T4 DNA ligase (NEB). The resulting construct (ca. 1 kb) was cloned into pCR2.1-TOPO with a TOPO TA Cloning Kit (Invitrogen, Carlsbad, CA, USA). The spectinomycin resistance cassette with *AvrII* sites at both ends was amplified with pRG5 (Kim et al., 2005) as a template. The spectinomycin resistance cassette was *AvrII*-digested and ligated into the *AvrII* site located between the flanking regions of the genes to be deleted. Plasmids bearing the mutant alleles were linearized by restriction enzyme digestion. The linearized plasmids were electroporated into *G. metallireducens* as described previously (Tremblay et al., 2012). Genotypes of the mutant strains were confirmed with PCR and the absence of undesired mutations was confirmed with Sanger sequencing.

## Results and discussion

In order to identify genes that might be specifically associated with the initial conversion of benzene to phenol, gene transcript abundance was compared in cells metabolizing benzene and cells metabolizing phenol. There were 11 genes with higher transcript abundance in cells metabolizing benzene than in cells metabolizing phenol (Table 1). Each of these genes was also more highly expressed in cells metabolizing benzene than in cells metabolizing benzoate, toluene, or acetate (Table 1).

**Table 1:** **Genes up-regulated at least two fold in *G. metallireducens* when benzene is the electron donor (*P*-value cutoff ≤ 0.1; NCBI GEO under accession number GSE33794)**.

**Gene**	**Annotation**	**Name**	**Fold change: benzene vs**.			
			**Phenol**	**Benzoate**	**Toluene**	**Acetate**
Gmet 0232^a^	Conserved hypothetical protein		3	20.2	2.6	18.1
Gmet 0244^a^	Conserved hypothetical protein		3.9	14	5	19.5
Gmet 0802	Putative porin		2.4	9.9	3.5	14.9
Gmet 1065	CRISPR-associated protein	*casD*	2.3	13.3	2.6	13
Gmet 2410^a^	Superoxide dismutase	*sodA*	6.1	2.3	2.7	4.3
Gmet 2833^a^	Conserved hypothetical protein		2.7	5.5	3	5.1
Gmet 3104	Flagellar operon protein of unknown function DUF3766		3.2	8.4	9	8
Gmet 3105	flagellar hook capping protein	*flgD*	2.5	5.9	7.4	5.4
Gmet 3076	Toxin, PIN family		2.7	12.4	3.4	9.5
Gmet 3229^a^	Thioredoxin-related protein disulfide reductase, putative		2.1	6.5	2.9	4.4
Gmet 3376^a^	Conserved hypothetical protein		2.2	137.3	3.6	141.6

a Genes studied by functional genetic.

Five of these genes with higher transcript abundance in benzene-metabolizing cells were predicted to be involved either in flagella synthesis, in the CRISPR system, or to be a toxin or a porin (Table 1), suggesting that they are unlikely to be involved in an enzymatic attack on benzene. Therefore, the function of these genes was not further investigated. Strains in which one of the remaining six genes was deleted were constructed to determine whether the loss of the gene specifically impacted benzene metabolism.

**Figure 1:**
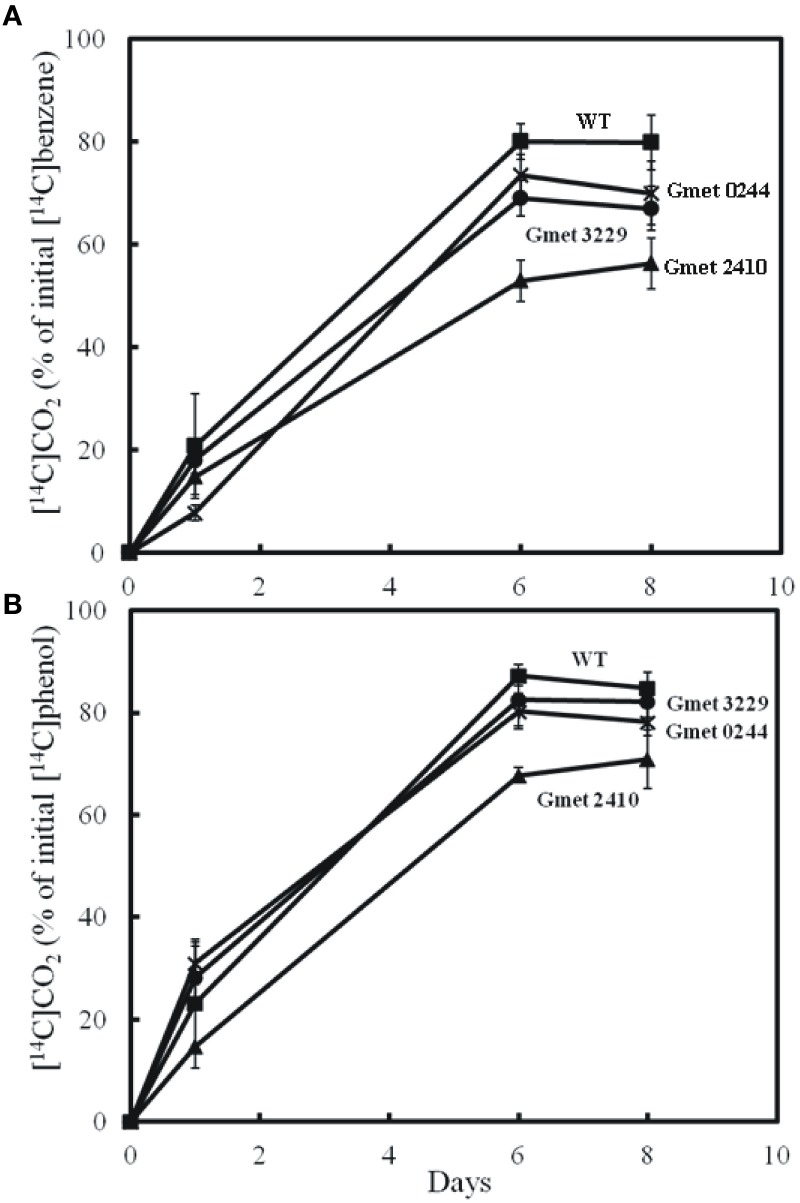
**Production of ^14^CO_2_ by cell suspension of strains lacking Gmet 0244, Gmet 2410, or Gmet 3229 from (A) [^14^C]benzene or (B) [^14^C]phenol**. The results are the mean and standard deviation for triplicate cell suspensions.

**Figure 2:**
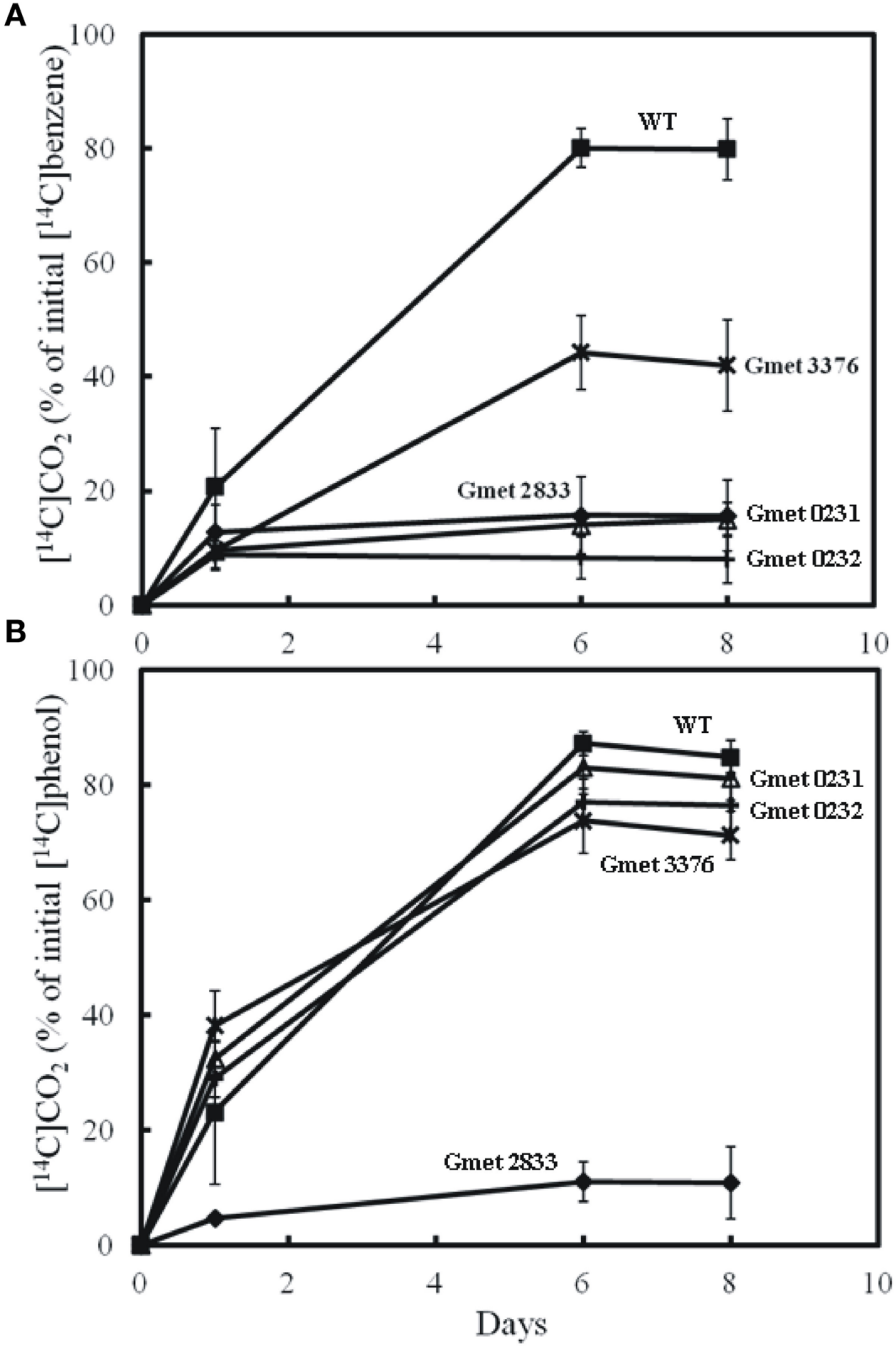
**Production of ^14^CO_2_ by cell suspension of strains lacking Gmet 0231, Gmet 0232, Gmet 2833, or Gmet 3376 from (A) [^14^C]benzene or (B) [^14^C]phenol**. The results are the mean and standard deviation for triplicate cell suspensions.

Strains lacking either Gmet 0244, Gmet 2410, or Gmet 3229, oxidized benzene at rates just slightly lower or comparable to wild type (Figure 1), suggesting that they did not encode enzymes important in benzene metabolism. Gmet 0244 encodes a hypothetical protein, whereas Gmet 2410 and Gmet 3229 are predicted to encode a superoxide dismutase, and a thioredoxin-related protein, respectively. The strain in which Gmet 2833 was deleted was deficient in phenol metabolism as well as benzene metabolism (Figure 2), indicating that this gene of unknown function did not have a role specific to benzene metabolism.

The strain lacking a functional Gmet 3376 oxidized benzene slightly slower and to a lesser extent than wild-type, whereas this strain metabolized phenol just as well as wild-type (Figure 2). Gmet 3376 encodes a hypothetical protein of 219 amino acids with no known homolog in any other *Geobacter* species. The ability of the Gmet 3376-deficient strain to continue to metabolize benzene at substantial rate suggests that it does not encode an enzyme that is required for benzene activation.

In contrast, deletion of the monocistronic gene Gmet 0232 specifically inhibited the metabolism of benzene, but not phenol (Figure 2). Gmet 0232 encodes for a hypothetical protein of 281 amino acids with homologs found only in *Geobacteraceae* family members and in *Nitrosomonas* sp. (strain *Is79A3*).

**Figure 3:**
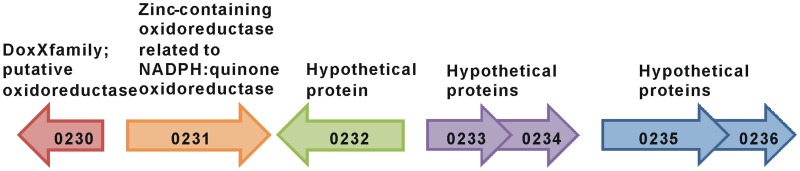
**The Gmet 0232 genomic region**.

The transcript abundance of the adjacent gene, Gmet 0231 (Figure 3), was elevated in benzene-metabolizing cells (44.6-fold compared to acetate-metabolizing cells; *P*-value = 0.00004), but the difference in transcript abundance between benzene- and phenol-metabolizing cells was too low to be considered to be significantly higher in benzene-metabolizing cells (1.7-fold higher; *P*-value = 0.003). Gmet 0231 is predicted to be a monocistronic gene encoding a zinc-containing oxidoreductase of the NADPH:quinone oxidoreductase family. A strain in which Gmet 0231 was deleted had a phenotype similar to the strain deficient in Gmet 0232; phenol was metabolized as well as in wild-type, but benzene metabolism was inhibited (Figure 2).

## Implications

The results demonstrate that *G. metallireducens* specifically requires genes Gmet 0231 and 0232 for the metabolism of benzene, but not phenol. This raises the possibility that Gmet 0231 or Gmet 0232, or both, encode an enzyme(s) involved in the initial activation of benzene to produce phenol, or serve an important ancillary role in this phase of benzene metabolism.

Gmet 0232 homologs are found in two *Geobacter* species, *Geobacter sulfurruducens* (Caccavo et al., 1994), and *Geobacter uraniireducens* (Shelobolina et al., 2008), that are unable to anaerobically oxidize monoaromatic hydrocarbons. Gmet 0231 homologs are found in multiple species throughout the three kingdoms of life that are not known to degrade monoaromatic hydrocarbons, including several *Geobacter* species. However, the co-localization of Gmet 0231 and Gmet 0232 found in *G. metallireducens* has not been observed in any other microbial genome sequence, suggesting that a functional relation between the proteins encoded by Gmet 0231 and Gmet 0232 might be necessary for benzene conversion to phenol by *G. metallireducens*. Now that Gmet 0231 and Gmet 0232 have been identified as having an important role in the initial steps in benzene metabolism, biochemical studies of the gene products is warranted.

Also warranted are studies on the function of genes localized near Gmet 0231 and Gmet 0232 in the *G. metallireducens* genome. For example, Gmet 0230 is predicted to encode an oxidoreductase of the DoxX family, which has sequence similarities with DoxD, a subunit of the membrane-bound thiosulphate:quinone oxidoreductase (Muller et al., 2004). Genes adjacent to Gmet 0232 are predicted to encode hypothetical proteins of unknown function (Figure 3), suggesting that genetic analysis of their function in benzene metabolism may also be warranted.

It has been suggested (Coates et al., 2002) that enzymes with functions similar to the ethylbenzene dehydrogenase found in *Aromataleum aromaticum* (Johnson and Spormann, 1999; Kniemeyer and Heider, 2001) or the acetylene hydratase of *Pelobacter acetylinus* (Schink, 1985; Rosner and Schink, 1995) might be involved in the anaerobic hydroxylation of benzene. None of the genes investigated here have any homology to the components of either of those enzyme complexes. Thus, these results suggest that *G. metallireducens* may possess a novel biochemistry for the anaerobic hydroxylation of benzene.

### Conflict of interest statement

The authors declare that the research was conducted in the absence of any commercial or financial relationships that could be construed as a potential conflict of interest.

## Supplementary material

Table S1 **Bacterial strains and plasmids used in this study**.

## References

[B1] Abu Laban N. Selesi D. Rattei T. Tischler P. Meckenstock R. U. (2010). Identification of enzymes involved in anaerobic benzene degradation by a strictly anaerobic iron-reducing enrichment culture. Environ. Microbiol. 12, 2783–2796 10.1111/j.1462-2920.2010.02248.x 20545743

[B2] Anderson R. T. Lovley D. R. (2000). Anaerobic bioremediation of benzene under sulfate-reducing conditions in a petroleum-contaminated aquifer. Environ. Sci. Technol. 34, 2261–2266 10.1021/es991211a

[B3] Caccavo F. Jr. Lonergan D. J. Lovley D. R. Davis M. Stolz J. F. McInerney M. J. (1994). *Geobacter sulfurreducens* sp. nov., a hydrogen- and acetate-oxidizing dissimilatory metal-reducing microorganism. Appl. Environ. Microbiol. 60, 3752–3759 7527204 10.1128/aem.60.10.3752-3759.1994 PMC201883

[B4] Caldwell M. E. Suflita J. M. (2000). Detection of phenol and benzoate as intermediates of anaerobic benzene biodegradation under different terminal electron-accepting conditions. Environ. Sci. Technol. 34, 1216–1220 10.1021/es990849j7527204

[B5] Chakraborty R. Coates J. D. (2005). Hydroxylation and carboxylation-two crucial steps of anaerobic benzene degradation by *Dechloromonas* strain RCB. Appl. Environ. Microbiol. 71, 5427–5432 10.1128/AEM.71.9.5427-5432.2005 16151134 PMC1214610

[B6] Chakraborty R. O'Connor S. M. Chan E. Coates J. D. (2005). Anaerobic degradation of benzene, toluene, ethylbenzene, and xylene compounds by *Dechloromonas* strain RCB. Appl. Environ. Microbiol. 71, 8649–8655 10.1128/AEM.71.12.8649-8655.2005 16332859 PMC1317370

[B7] Chaudhuri B. K. Wiesmann U. (1995). Enhanced anaerobic degradation of benzene by enrichment of mixed microbial culture and optimization of the culture medium. Appl. Microbiol. Biotechnol. 43, 178–187 10.1007/BF00170641 7766131

[B8] Coates J. D. Chakraborty R. Lack J. G. O'Connor S. M. Cole K. A. Bender K. S. (2001). Anaerobic benzene oxidation coupled to nitrate reduction in pure culture by two strains of *Dechloromonas*. Nature 411, 1039–1043 10.1038/35082545 11429602

[B9] Coates J. D. Chakraborty R. McInerney M. J. (2002). Anaerobic benzene biodegradation-a new era. Res. Microbiol. 153, 621–628 10.1016/S0923-2508(02)01378-5 12558180

[B10] Foght J. (2008). Anaerobic biodegradation of aromatic hydrocarbons: pathways and prospects. J. Mol. Microbiol. Biotechnol. 15, 93–120 10.1159/000121324 18685265

[B11] Grbic-Galic D. Vogel T. M. (1987). Transformation of toluene and benzene by mixed methanogenic cultures. Appl. Environ. Microbiol. 53, 254–260 3105454 10.1128/aem.53.2.254-260.1987 PMC203647

[B12] Head I. M. Larter S. R. Gray N. D. Sherry A. Adams J. J. Aitken C. M. (2010). Hydrocarbon degradation in petroleum reservoirs, in Handbook of Hydrocarbon and Lipid Microbiology, Vol. 4, eds Timmis K. N. McGenity T. van der Meer J. R. de Lorenzo V. (Heidelberg; Germany: Springer), 3097–3109

[B13] Heider J. Schühle K. (2013). Anaerobic biodegradation of hydrocarbons including methane, in The Prokaryotes, eds Rosenberg E. Delong E. F. Lory S. Stackebrandt E. Thompson F. (Berlin; Heidelberg: Springer-Verlag), 605–634 10.1007/978-3-642-30141-4_80

[B14] Holmes D. E. Risso C. Smith J. A. Lovley D. R. (2011). Anaerobic oxidation of benzene by the hyperthermophilic archaeon *Ferroglobus placidus*. Appl. Environ. Microbiol. 77, 5926–5933 10.1128/AEM.05452-11 21742914 PMC3165377

[B15] Johnson H. A. Pelletier D. A. Spormann A. M. (2001). Isolation and characterization of anaerobic ethylbenzene dehydrogenase, a novel Mo-Fe-S enzyme. J. Bacteriol. 183, 4536–4542 10.1128/JB.183.15.4536-4542.2001 11443088 PMC95348

[B16] Johnson H. A. Spormann A. M. (1999). *In vitro* studies on the initial reactions of anaerobic ethylbenzene mineralization. J. Bacteriol. 181, 5662–5668 10482506 10.1128/jb.181.18.5662-5668.1999 PMC94085

[B17] Kim B. C. Leang C. Ding Y. H. Glaven R. H. Coppi M. V. Lovley D. R. (2005). OmcF, a putative c-type monoheme outer membrane cytochrome required for the expression of other outer membrane cytochromes in *Geobacter sulfurreducens*. J. Bacteriol. 187, 4505–4513 10.1128/JB.187.13.4505-4513.2005 15968061 PMC1151787

[B18] Kniemeyer O. Heider J. (2001). Ethylbenzene dehydrogenase, a novel hydrocarbon-oxidizing molybdenum/iron-sulfur/heme enzyme. J. Biol. Chem. 276, 21381–21386 10.1074/jbc.M101679200 11294876

[B19] Kunapuli U. Griebler C. Beller H. R. Meckenstock R. U. (2008). Identification of intermediates formed during anaerobic benzene degradation by an iron-reducing enrichment culture. Environ. Microbiol. 10, 1703–1712 10.1111/j.1462-2920.2008.01588.x 18412549

[B20] Lovley D. R. (1991). Dissimilatory Fe(III) and Mn(IV) reduction. Microbiol. Rev. 55, 259–287 1886521 10.1128/mr.55.2.259-287.1991 PMC372814

[B21] Lovley D. R. (2000). Anaerobic benzene degradation. Biodegradation 11, 107–116 10.1023/A:1011191220463 11440238

[B22] Lovley D. R. Coates J. D. Woodward J. C. Phillips E. (1995). Benzene oxidation coupled to sulfate reduction. Appl. Environ. Microbiol. 61, 953–958 16534979 10.1128/aem.61.3.953-958.1995 PMC1388378

[B23] Lovley D. R. Giovannoni S. J. White D. C. Champine J. E. Phillips E. J. P. Gorby Y. A. (1993). *Geobacter metallireducens* gen. nov. sp. nov., a microorganism capable of coupling the complete oxidation of organic compounds to the reduction of iron and other metals. Arch. Microbiol. 159, 336–344 10.1007/BF00290916 8387263

[B24] Lovley D. R. Phillips E. J. (1988). Novel mode of microbial energy metabolism: organic carbon oxidation coupled to dissimilatory reduction of iron or manganese. Appl. Environ. Microbiol. 54, 1472–1480 16347658 10.1128/aem.54.6.1472-1480.1988 PMC202682

[B25] Lovley D. R. Ueki T. Zhang T. Malvankar N. S. Shrestha P. M. Flanagan K. (2011). *Geobacter*: the microbe electric's physiology, ecology, and practical applications. Adv. Microb. Physiol. 59, 1–100 10.1016/B978-0-12-387661-4.00004-5 22114840

[B26] Lovley D. R. Woodward J. C. Chapelle F. H. (1994). Stimulated anoxic biodegradation of aromatic hydrocarbons using Fe(III) ligands. Nature 370, 128–131 10.1038/370128a0 8022480

[B27] Lovley D. R. Woodward J. C. Chapelle F. H. (1996). Rapid anaerobic benzene oxidation with a variety of chelated Fe(III) forms. Appl. Environ. Microbiol. 62, 288–291 16535218 10.1128/aem.62.1.288-291.1996 PMC1388759

[B28] Masumoto H. Kurisu F. Kasuga I. Tourlousse D. M. Furumai H. (2012). Complete mineralization of benzene by a methanogenic enrichment culture and effect of putative metabolites on the degradation. Chemosphere 86, 822–888 10.1016/j.chemosphere.2011.11.051 22205046

[B29] Meckenstock R. U. Mouttaki H. (2011). Anaerobic degradation of non-substituted aromatic hydrocarbons. Curr. Opin. Biotechnol. 22, 406–414 10.1016/j.copbio.2011.02.009 21398107

[B30] Muller F. H. Bandeiras T. M. Urich T. Teixeira M. Gomes C. M. Kletzin A. (2004). Coupling of the pathway of sulphur oxidation to dioxygen reduction: characterization of a novel membrane-bound thiosulphate:quinone oxidoreductase. Mol. Microbiol. 53, 1147–1160 10.1111/j.1365-2958.2004.04193.x 15306018

[B31] Oberender J. Kung J. W. Seifert J. von Bergen M. Boll M. (2012). Identification and characterization of a succinyl-coenzyme A (CoA):benzoate CoA transferase in *Geobacter metallireducens*. J. Bacteriol. 194, 2501–2508 10.1128/JB.00306-12 22408161 PMC3347176

[B32] Rosner B. M. Schink B. (1995). Purification and characterization of acetylene hydratase of *Pelobacter acetylenicus*, a tungsten iron-sulfur protein. J. Bacteriol. 177, 5767–5772 7592321 10.1128/jb.177.20.5767-5772.1995 PMC177396

[B33] Sakai N. Kurisu F. Yagi O. Nakajima F. Yamamoto K. (2009). Identification of putative benzene-degrading bacteria in methanogenic enrichment cultures. J. Biosci. Bioeng. 108, 501–507 10.1016/j.jbiosc.2009.06.005 19914583

[B34] Salinero K. K. Keller K. Feil W. S. Feil H. Trong S. di Bartolo G. (2009). Metabolic analysis of the soil microbe *Dechloromonas aromatica* str. RCB: indications of a surprisingly complex life-style and cryptic anaerobic pathways for aromatic degradation. BMC Genomics 10:351 10.1186/1471-2164-10-351 19650930 PMC2907700

[B35] Schink B. (1985). Fermentation of acetylene by an obligate anaerobe, *Pelobacter acetylenicus* sp. nov. Arch. Microbiol. 142, 295–301 10.1007/BF00693407

[B36] Shelobolina E. S. Vrionis H. A. Findlay R. H. Lovley D. R. (2008). *Geobacter uraniireducens* sp. nov., isolated from subsurface sediment undergoing uranium bioremediation. Int. J. Syst. Evol. Microbiol. 58, 1075–1078 10.1099/ijs.0.65377-0 18450691

[B37] Smith J. A. Lovley D. R. Tremblay P. L. (2013). Outer cell surface components essential for Fe(III) oxide reduction by *Geobacter metallireducens*. Appl. Environ. Microbiol. 79, 901–907 10.1128/AEM.02954-12 23183974 PMC3568551

[B38] Thauer R. K. Jungermann K. Decker K. (1977). Energy conservation in chemotrophic anaerobic bacteria. Bacteriol. Rev. 41, 100–180 860983 10.1128/br.41.1.100-180.1977 PMC413997

[B39] Tremblay P. L. Aklujkar M. Leang C. Nevin K. P. Lovley D. (2012). A genetic system for *Geobacter metallireducens*: role of the flagellin and pilin in the reduction of Fe(III) oxide. Environ. Microbiol. Rep. 4, 82–88 10.1111/j.1758-2229.2011.00305.x 23757233

[B41] Ulrich A. C. Beller H. R. Edwards E. A. (2005). Metabolites detected during biodegradation of ^13^C_6_-benzene in nitrate-reducing and methanogenic enrichment cultures. Environ. Sci. Technol. 39, 6681–6691 10.1021/es050294u 16190227

[B42] van der Zaan B. M. Saia F. T. Stams A. J. Plugge C. M. de Vos W. M. Smidt H. (2012). Anaerobic benzene degradation under denitrifying conditions: peptococcaceae as dominant benzene degraders and evidence for a syntrophic process. Environ. Microbiol. 14, 1171–1181 10.1111/j.1462-2920.2012.02697.x 22296107

[B43] Vogel T. M. Grbicgalic D. (1986). Incorporation of oxygen from water into toluene and benzene during anaerobic fermentative transformation. Appl. Environ. Microbiol. 52, 200–202 16347109 10.1128/aem.52.1.200-202.1986 PMC203449

[B44] Vogt C. Kleinsteuber S. Richnow H. H. (2011). Anaerobic benzene degradation by bacteria. Microb. Biotechnol. 4, 710–724 10.1111/j.1751-7915.2011.00260.x 21450012 PMC3815408

[B45] Weelink S. A. B. van Eekert M. H. A. Stams A. J. M. (2010). Degradation of BTEX by anaerobic bacteria: physiology and application. Rev. Environ. Sci. Biotechnol. 9, 359–385 10.1007/s11157-010-9219-29572976

[B46] Weiner J. M. Lovley D. R. (1998). Rapid benzene degradation in methanogenic sediments from a petroleum-contaminated aquifer. Appl. Environ. Microbiol. 64, 1937–1939 9572976 10.1128/aem.64.5.1937-1939.1998 PMC106255

[B47] Widdel F. Rabus R. (2001). Anaerobic biodegradation of saturated and aromatic hydrocarbons. Curr. Opin. Biotechnol. 12, 259–276 10.1016/S0958-1669(00)00209-3 11404104

[B48] Zhang T. Bain T. S. Nevin K. P. Barlett M. A. Lovley D. R. (2012). Anaerobic benzene oxidation by *Geobacter* species. Appl. Environ. Microbiol. 78, 8304–8310 10.1128/AEM.02469-12 23001648 PMC3497359

[B49] Zhang T. Gannon S. M. Nevin K. P. Franks A. E. Lovley D. R. (2010). Stimulating the anaerobic degradation of aromatic hydrocarbons in contaminated sediments by providing an electrode as the electron acceptor. Environ. Microbiol. 12, 1011–1020 10.1111/j.1462-2920.2009.02145.x 20105223

[B50] Zhang T. Tremblay P. L. Chaurasia A. K. Smith J. A. Bain T. S. Lovley D. R. (2013). Anaerobic benzene oxidation via phenol in *Geobacter metallireducens*. Appl. Environ. Microbiol. 79, 7800–7806 10.1128/AEM.03134-13 24096430 PMC3837793

