# Supplementary material for: Identification of genes specifically required for the anaerobic metabolism of benzene in Geobacter metallireducens
Source: Front Microbiol. 2014 May 22;5:245. doi: 10.3389/fmicb.2014.00245 (PMC4033198; doi:10.3389/fmicb.2014.00245)
Supplement: Table S1 — Bacterial strains and plasmids used in this study. [file DataSheet1.DOCX]

| TABLE S1. Bacterial strains and plasmids used in this study | | |
| --- | --- | --- |
| Strain or plasmid | Relevant characteristic(s) | Source or reference |
| Strains |  |  |
| *E. coli* |  |  |
| TOP10 | *recA1 endA1 gyrA96 thi-1 hsdR17* (r_K_^–^ m_K_^+^) *supE44 relA1* ∆*lacU169* | Invitrogen, Carlsbad, CA |
|  |  |  |
| *G. metallireducens* |  |  |
| wild type (DSM 7210) | wild type | Lovley and Lonergan,1990 |
| ppsA | *ΔppsA ::Sp^r^* | This work |
| ppcB | Δ*ppcB::Sp^r^* | This work |
|  |  |  |
| Plasmids |  |  |
| pCR2.1-TOPO | PCR cloning vector; Ap^r^, Km^r^ | Invitrogen, Carlsbad, CA |
| pRG5 | Broad-host-range taclac expression vector; Sp^r^ | Kim *et al*., 2005 |
| pCR2.1Gmet 0231up5’+3’dn | pCR2.1TOPO carrying Gmet 0231up5’+3’dn; Ap^r^, Km^r^ | This work |
| pCR2.1ΔGmet 0231*::Sp^r^* | pCR2.1TOPO carrying ΔGmet 0231*::Sp^r^*; Ap^r^, Km^r^ | This work |
| pCR2.1Gmet 0232up5’+3’dn | pCR2.1TOPO carrying Gmet 0232up5’+3’dn; Ap^r^, Km^r^ | This work |
| pCR2.1ΔGmet 0232*::Sp^r^* | pCR2.1TOPO carrying ΔGmet 0232*::Sp^r^*; Ap^r^, Km^r^ | This work |
| pCR2.1Gmet 0244up5’+3’dn | pCR2.1TOPO carrying Gmet 0244up5’+3’dn; Ap^r^, Km^r^ | This work |
| pCR2.1ΔGmet 0244*::Sp^r^* | pCR2.1TOPO carrying ΔGmet 0244*::Sp^r^*; Ap^r^, Km^r^ | This work |
| pCR2.1Gmet 2410up5’+3’dn | pCR2.1TOPO carrying Gmet 2410up5’+3’dn; Ap^r^, Km^r^ | This work |
| pCR2.1ΔGmet 2410*::Sp^r^* | pCR2.1TOPO carrying ΔGmet 2410*::Sp^r^*; Ap^r^, Km^r^ | This work |
| pCR2.1Gmet 2833up5’+3’dn | pCR2.1TOPO carrying Gmet 2833up5’+3’dn; Ap^r^, Km^r^ | This work |
| pCR2.1ΔGmet 2833*::Sp^r^* | pCR2.1TOPO carrying ΔGmet 2833*::Sp^r^*; Ap^r^, Km^r^ | This work |
| pCR2.1Gmet 3229up5’+3’dn | pCR2.1TOPO carrying Gmet 3229up5’+3’dn; Ap^r^, Km^r^ | This work |
| pCR2.1ΔGmet 3229*::Sp^r^* | pCR2.1TOPO carrying ΔGmet 3229*::Sp^r^*; Ap^r^, Km^r^ | This work |
| pCR2.1Gmet 3376up5’+3’dn | pCR2.1TOPO carrying Gmet 3376up5’+3’dn; Ap^r^, Km^r^ | This work |
| pCR2.1ΔGmet 3376*::Sp^r^* | pCR2.1TOPO carrying ΔGmet 3376*::Sp^r^*; Ap^r^, Km^r^ | This work |
